# Investigating the obesogenic effects of marketing snacks with toys: an experimental study in Latin America

**DOI:** 10.1186/1475-2891-12-95

**Published:** 2013-07-10

**Authors:** Dario Gregori, Simonetta Ballali, Claudia Elena Gafare, Adriana Casella, Giulia Stefanini, Rogenia de Sousa Alves, Laura Franchin, Ignacio Amador, Neila Maria Almedia Da Silva, Javier Dibildox

**Affiliations:** 1 Department of Cardiac, Tharacic and Vascular Science, Unit of Biostatisticsm, Epidemiology and Public Health, Via Loredan, 18, 35121, Padova, Italy; 2 Prochild ONLUS, Trieste, Italy; 3 Department of Nutrition, University of Buenos Aires and Food and Diet Therapy Service, Acute General Hospital Juan A. Fernández, Buenos Aires, Argentina; 4 Ramos Mejia Psychology Center, Buenos Aires, Argentina; 5 ZETA Research Ltd., Trieste, Italy; 6 Instituto Cuarto Centenario, San Louis, Potosì, Mexico; 7 Escola Municipal de Ensino Infantil e Fundamental Odilon Braveza, Fortaleza, Brazil; 8 University San Louis Potosì, San Louis, Potosì, Mexico

**Keywords:** Toys for marketing food, Obesogenic environment, TV exposure, TV advertising, *Ad libitum* eating studies

## Abstract

**Background:**

The inclusion of toys in food packages is a common marketing practice, and it is suspected of promoting obesogenic behaviours. This study aimed to determine whether toys packaged with food are indeed increasing the amount of food eaten by children, and if this effect is enhanced by contemporary exposure to TV and/or advertising.

**Methods:**

A total of 600 children (balanced according to gender and age groups, 3–6 and 7–10 years old) were randomized in three school facilities in Argentina, Brazil and Mexico and exposed to food (snacks) alone or food associated with toys in an experimental setting. All of the children received the same meal at lunchtime. The products were packages in which chocolate was associated with toys in an egg-shaped container partially filled by chocolate. The children were asked to eat *ad libitum* for 20 minutes during the afternoon break. In addition, the children were randomized into two groups and either shown or not shown a movie cartoon, with three different levels of exposure to commercials in the TV viewing condition (1, 2 or 3 advertisements).

**Results:**

No significant differences emerged between the “toys” and “no toys” groups even after taking into account exposure to TV, commercials and other confounding factors.

**Conclusions:**

The inclusion of toys in food packages was not shown *per se* to lead to an increase in the caloric intake of children.

## Introduction

There is growing concern regarding the rapidly increasing rate of obesity in childhood, which has become epidemic in some areas and is on the rise in others, with an estimated 17.6 million obese children worldwide; particularly, there are increasing trends in emerging economies. The early onset of obesity, which is a major risk factor for a number of non-communicable diseases, including diabetes mellitus, coronary heart disease, hypertension and some forms of cancer, needs to be addressed with high-impact strategies for prevention and, where this fails, with clinical management [1-3]. Specific attention has recently focused on limiting inappropriate (in amount and typology) caloric intake within a context defined as an “obesogenic environment” of childhood, which is an environment where TV viewing, advertising, marketing strategies promoting food associated with gadgets and snacking are identified as possible risk factors [4-7].

Recent literature and scientific research has therefore focused on television and advertising and assessed their influence on children’s eating behaviours, with results showing an increased daily caloric intake. Ample studies investigating the effects of TV viewing on total energy intake have concluded that there is a positive effect of increased screen time on energy intake in children and adolescents [8,9]. Other studies have considered the short- and long-term effects of advertising on children’s food intake and the promotion of unhealthy diets [4,10], demonstrating a causal link between food consumption and television exposure and advertising [7]. Snacking during screen time is often associated with high, energy-dense products, therefore undermining the healthy balance of energy intake [11,12]. In addition, other studies suggest the existence of a specific gender effect in the sense that boys seem to be more susceptible to food cues than girls [13]. There is also growing evidence that children with greater exposure to commercials appear to be more responsive to food promotion messages than children with lower previous advertising exposure [14].

In this context, a long-standing yet under-investigated factor that might influence energy intake is the promotion of food to children in association with gadgets, usually toys, aimed at capturing the attention of the child and increasing loyalty to the brand or product; this could be related to obesogenic effects [12]. Considering the possible link between gadgets and an obesogenic effect, a recent ordinance in the U.S.A. was implemented to prohibit the distribution of toys and other incentives to children in conjunction with meals, foods, or beverages that do not meet minimal nutritional criteria [15].

The debate over such regulatory measures is ongoing in several other countries as well, both in established and emerging economies. In Latin America, where obesity is an emerging trend, particularly in children, the need for regulatory proposals grounded on strong evidence has faced a lack of experimental information on the behavioural factors related to obesity. In such geographical areas, decision makers often refer to evidence translated from experimental studies that are usually performed in North American or, more generally, that refer to an Anglo-Saxon cultural setting [4,5]. Such studies are indeed designed to detect an excess of caloric intake under certain experimental conditions, particularly exposure to TV watching and snacking, advertising or branding [4,5,10,12,16].

To fill this gap, a wide experimental study has been conducted in three Latin American countries (Mexico, Brazil and Argentina) that are facing a steep growth in obesity rates in children (with obesity rates of 41.8% [17], 22.1% [18] and 19.3%, respectively [19]). The primary aim of this study was to assess whether including toys in food packages increases the amount of food eaten by children during a snacking occasion.

Given the abovementioned evidence of the role of TV and advertising in promoting an excessive consumption of (branded) food in children [20], the children were also exposed to TV and advertising during the experimental sessions, to adjust for both their potential confounding effects and their intrinsic relevance as intervening factors.

This resulted in a factorial study design, which, at the price of a certain logistical and analytical complexity, aimed in a controlled situation to evaluate the effects on the amount of snacks eaten by the children when the children (*i*) had food presented with toys in the package, (*ii*) were exposed to TV when snacking, (*iii*) were exposed to advertising (at an increasing intensity during TV exposure) and (*iv*) had potential interactions from a combination of all these factors.

## Materials and methods

### Design

The experimental study was a 2x5 full factorial *ad libitum* eating design. The first factor was represented by exposure to the toy during the experimental session and was organized on two levels: “food with toy” (Toy) or “food alone” (NoToy). The second factor was exposure to TV and advertising during the experimental session and was organized in five levels: “no exposure to TV” (NoFilmNoSpot), “exposure to TV without advertising” (FilmNoSpot), “exposure to TV and one advertisement” (FilmLowSpot), “exposure to TV and two advertisements” (FilmMediumSpot) and “exposure to TV and three advertisements” (FilmHighSpot).

The sample size was computed with reference to an alpha equal to 0.05 and a power of 0.90, which was aimed at detecting at least a difference of 20 Kcal of caloric intake (assuming an equal standard deviation in the two groups of approximately 10 Kcal) between the two groups “food with toy” (Toy) and “food alone” (NoToy) in each of the 10 randomization cells.

### Participants

Six-hundred children were estimated to be necessary to accomplish this study’s goals. The sample was equally balanced between males and females ranging from 3 to 10 years old. Children were studied in 3 different Latin American countries: Argentina (120 children), Brazil (360 children) and Mexico (120 children). Children were enrolled in a school setting in each of the countries involved in the study, all of which were in metropolitan areas: Buenos Aires (Argentina), Fortaleza (Brazil) and San Louis Potosí (Mexico). The schools were of a middle socio-economic level. Limited percentages of the children had previously been exposed to the experimental product either via advertising or by personal experience (between 5% in Brazil and 10% in Mexico). Children with cognitive disorders or metabolic diseases or allergies to the products offered during the experimental session were excluded. Parental informed consent was obtained for all children prior to each child’s participation in the study. Participant treatment followed the experimental and ethical guidelines issued by the American Psychologist Association. Appropriate permission was obtained by the Institutional Review Boards.

### Randomization Procedure

Each child was randomized to one of the 10 cells of the full-factorial design, and the randomization was administered by ad hoc software integrated with the data collection and study conduction software system. The randomization was subdivided by age (two groups of children 3–6 and 7–10 years old) and by gender (male and female) to ensure a complete balance of the two potential confounding factors.

The children were evaluated during the afternoon break in a quiet room within the school buildings. The schools had a consistent pattern of programmed physical activity, and they were instructed to give the same meal to all the children participating in the study. The parents were asked not to assist during the session; if they refused, they were required to sit at a rear position in order to fill out the parent questionnaires without being seen by the children, who were sitting at a table facing a monitor (if applicable, according to the group to which they had been randomized). The children were video-recorded through two hidden HD cameras positioned to record frontal and side images, while the investigator assumed a back-screen position to easily accommodate the children’s requests without putting any pressure on the children’s performances.

The film was a non-spoken (but not silent) cartoon lasting approximately 22 minutes. The choice of this type of cartoon was made in order to avoid any language bias that might have arisen based on the multicultural sample (the children spoke Portuguese in Brazil and Spanish in Mexico and Argentina, although with different accents). The principal protagonist was the Disney character Pluto©. The movie consisted of a set of episodes, in accordance with previous studies [7,21]. Following the setting adopted in other studies [7], the commercials referred to the product given to the children and lasted 30 seconds and were in the country-specific language. Every commercial presented during the movie was different from the previous one with a maximum of three different commercials for the children randomized to that specific group.

**Figure 1:**
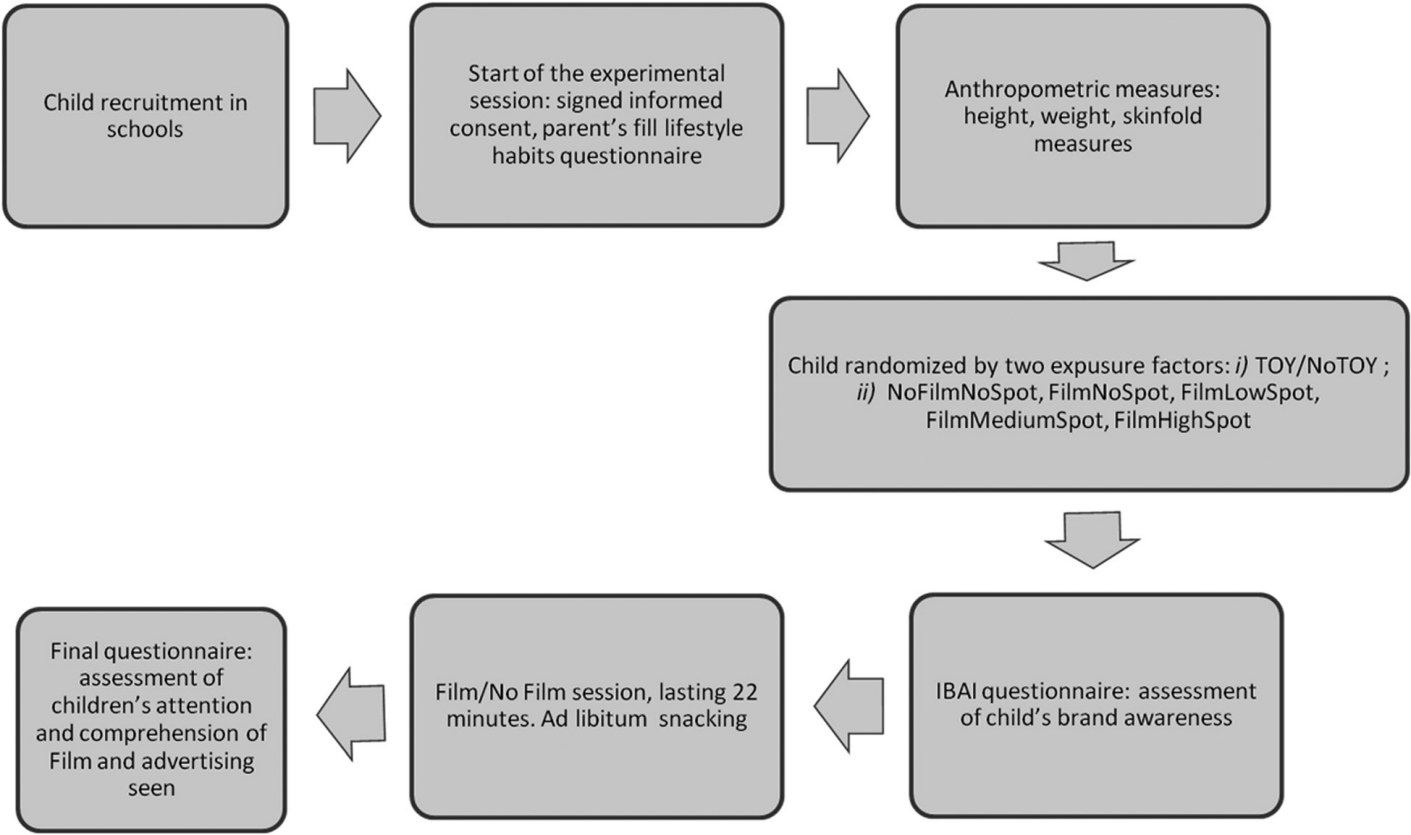
Flow-chart for the conduction of the study.

The children were evaluated at the first meeting in order to record their BMI and basic characteristics. At the second meeting, the IBAI (International Brand Awareness Instrument) questionnaire [22] (short version) was administered to the children by the interviewer. When the IBAI assessment was fully completed, the interviewer explained the progression of the study to the child. All of the steps are presented in Figure 1.

The snack provided for the study was a chocolate-based product mimicking an Easter egg, characterized by the presence of food (chocolate) on one side and a small toy on the other. The product, a standard commercial product available in Europe and other countries, was chosen for two reasons: (*i*) to evaluate the influence of the toys, it was necessary to have a product with various types of toys combined routinely with the snack in a single, easy-to-handle product, and (*ii*) the particular selling combination of the snack allowed for precise weighing of the contents, both before the study in order to assess the starting point and after the child had eaten, giving an accurate estimate of the number of calories consumed by every child. The nutritional characteristics of the products are presented in Table 1.

**Table 1:** Nutritional composition of the egg-shaped products

**Nutritional facts**	**Egg-shaped snack (100 g)**	**Egg-shaped snack (20 g)**
Energy (kcal)	545	109
Proteins (g)	8.3	1.7
Carbohydrates (g)	55	11
Sugar (g)	51	10.2
Fats (g)	32.4	6.5
Saturated (g)	14	2.8
Fibre (g)	1.8	0.4
Sodium (g)	0.083	0.017
Vitamin E (mg)	3.5 (35% RDA**)	0.7 (7% RDA**)
Vitamin B2 (mg)	0.29 (18% RDA**)	0.05 (3% RDA**)
Vitamin B12 (μg)	0.40 (40% RDA**)	0.08 (8%RDA**)
Calcium (mg)	210 (26% RDA**)	42 (5% RDA**)
Phosphorous (mg)	200 (25% RDA**)	40 (5% RDA**)
Magnesium (mg)	48 (16% RDA**)	9 (3% RDA**)

Data are provided per 100 g and per portion (which is a 20 g snack).

The half of the snack containing the chocolate was weighed and then offered alone to the children randomized to the NoToy group, whereas in the Toy group, the chocolate was given combined with the part containing the toy. In both cases the same snack was thus offered, for an amount of 109 Kcal each. If the child was assigned to a TV-exposure group, the movie was started at the time of the first offering of the snack to the child and not interrupted for any reason. After the first snack, the experimenter was allowed to present additional snacks, one at a time (always waiting for the child’s request). Each child was allowed to eat *ad libitum* up to a maximum of 12 snacks over the 22-minute time slot. The offering of the snack was performed using a pre-developed protocol as a way to control the influence of the investigator on the child as much as possible. The protocol specified the way the investigator was supposed to interact with the child both verbally (i.e.: smiling, kindly asking but not pushing for additional snacks, …) and by gestural approaches (i.e.: how far from the child the snack was offered, in which position with respect to her/his eyes, …).

Once the experimental session ended, the experimenter weighed the chocolate residues for each product, recording all weights in the study software. All of the sessions were digitally recorded for subsequent examination and data quality assurance.

### Measures

#### Socio-Demographic variables

During the first meeting, the basic characteristics of the children were recorded by the study conductors. The children were weighed and measured in light clothing and without shoes on an electronic stand-up balance scale and with a rigid metric belt. The measurements were taken with the child’s back against the wall with the back of the feet touching the wall and with a straight angle formed between the wall and the floor. A straight surface was then placed over the child’s head, and a mark was fixed on the wall, which was the initial point. Weights and heights were utilized to calculate the BMI values, according to CDC Pediatric Growth Charts [23]. The BMI z-scores were computed for each child [24]. Additional information on the presence of any allergy was recorded during the first meeting.

The questionnaire given to the child’s parents was divided into two sections. The first section was aimed at determining the socio-demographic characteristics of the entire family. It included questions about the parental educational level, the BMI of the parents and siblings, and a detailed set of queries covering the principal meals and basic physical activities performed within the family. Questions on the child’s eating habits were introduced in the second part of the questionnaire to assess the TV viewing habits and the physical activity habits of the children and their families.

### Brand awareness

A validated questionnaire to assess the child’s brand awareness, the IBAI questionnaire [22], was utilized in this study [22]. The questionnaire was composed of 12 images of food products on the market, including both international and country-specific products. The children were asked to recognize the brand by matching it to an image chosen from 4 different options. At the end, the interviewer questioned the child on the specific name of the product. Brand Awareness Scores (IBAI-score) could range from a minimum of 0 to a maximum of 36 points. A cut-off value of 16 points was used to define two groups: low brand awareness children (<16) and high brand awareness children (≥ 16) [22].

### Snack weighting

At the beginning of the study, every snack was assigned with a specific code and weighed in order to record the data within the software used for the study. At the end of every session, each snack eaten by the child was weighed once again to calculate the weight difference in terms of the amount eaten. All weights were collected by the mean of an Acculab© precision weighing scale with the capacity of 510 g and 0.1 g readability.

### Statistical analysis

A basic exploratory data analysis was performed on the sample and was reported using the median (I-III quartile) for continuous variables and the percentages (absolute numbers) for categorical variables, as appropriate.

The main analysis was based on a linear model where the subdivided factors (i.e., Gender and Age in two classes according to the randomization procedure, Toy, FilmSpot and the interaction between FilmSpot and Toy) have been inserted in the model. This was the base model used in the analysis. Specific investigations on single factor-level effects were conducted using the appropriate linear contrasts.

To check for additional confounding factors, six models were developed. In each model, variables were added to the base model, leading to the following hierarchical models, indicated as Base, M1, M2, BA (Brand Awareness), M3, M4:

1. Base: Nation + Age + Gender + Toy + FilmSpot + Toy:FilmSpot

2. M1: Base + BMI + Breast Feed + Hours/Week TV + Physical activity (hours/week)

3. M2: M1 + BMI Father + BMI Mother + number Brothers + number of Sisters

4. BA: M2 + Brand Awareness (BA) IBAI-Score

5. M3: BA + Number of rooms in the house + number of TVs in the house + Educational level of the mother

6. M4: M3 + breakfast in the morning + fruit portions/day + vegetables portions/day

Each model was individually evaluated, selecting among the candidate variables by using the AIC criterion in a backward fashion, which forced the inclusion of the design variables in each of the models. Thus, for each model, the significance of the main experimental factors (Toy, FilmSpot and Toy-FilmSpot interaction) was assessed.

In addition, children were identified as “high consumers” if their caloric intake during the experimental session exceeded the top quartile of the observed distribution. The variables related to the probability of being a high consumer were modelled using a logistic regression model and selected via the AIC criterion in a backward fashion.

The analyses were performed using the R System [25].

## Results

### Sample characteristics

Characteristics of the sample are provided in Table 2.

**Table 2:** Description of the sample

**Sample characteristics**		**N**	**Argentina**	**Brazil**	**Mexico**	**Total**	**P-value**
			**(N = 120)**	**(N = 360)**	**(N = 120)**	**(N = 600)**	
Child BMI		600	14.91/16.43/19.01	14.99/16.35/18.12	14.88/16.40/18.79	14.91/16.37/18.35	p = 0.781
Mother BMI		485	21.49/23.04/26.15	22.31/25.66/28.77	23.32/24.65/25.54	22.22/24.54/27.48	p < 0.001
Father BMI		340	24.82/26.99/29.06	22.77/25.12/27.68	24.74/25.47/26.34	24.22/25.75/27.58	p < 0.001
Neonatal feeding	*Breast-feeding*	553	77% (90)	93% (299)	50% (57)	81% (446)	p < 0.001
	*Bottle-feeding*		12% (14)	6% (18)	26% (29)	11% (61)	
	*Mixed (breast + bottle)*		11% (13)	2% (6)	24% (27)	8% (46)	
Number of sisters	*0*	600	52% (62)	44% (159)	68% (82)	50% (303)	p < 0.001
	*1*		38% (46)	35% (125)	30% (36)	34% (207)	
	*2*		9% (11)	13% (46)	2% (2)	10% (59)	
	*3*		1% (1)	5% (18)	0% (0)	3% (19)	
	*4*		0% (0)	2% (7)	0% (0)	1% (7)	
	*5*		0% (0)	0% (1)	0% (0)	0% (1)	
	*6*		0% (0)	1% (4)	0% (0)	1% (4)	
Number of brothers	*0*	840	57% (69)	40% (144)	57% (69)	47% (282)	p < 0.001
	*1*		32% (39)	34% (122)	40% (48)	35% (209)	
	*2*		9% (11)	17% (60)	2% (2)	12% (73)	
	*3*		1% (1)	7% (26)	1% (1)	5% (28)	
	*4*		0% (0)	1% (5)	0% (0)	1% (5)	
	*5*		0% (0)	0% (1)	0% (0)	0% (3)	
	*6*		0% (0)	0% (1)	0% (0)	0% (1)	
	*8*		0% (0)	0% (1)	0% (0)	0% (1)	
Frequency of breakfast before school	*2-3 times per week*	600	5% (6)	5% (19)	4% (5)	5% (30)	p = 0.024
	*3-4 times per week*		8% (10)	4% (15)	12% (15)	7% (40)	
	*Never*		11% (13)	10% (37)	4% (5)	9% (55)	
	*Every day*		76% (91)	80% (289)	79% (95)	79% (475)	
Daily fruit portions	*1*	600	52% (63)	31% (111)	42% (51)	38% (225)	p < 0.001
	*2*		22% (27)	30% (107)	48% (57)	32% (191)	
	*3*		8% (9)	12% (42)	6% (7)	10% (58)	
	*4*		2% (2)	6% (20)	2% (2)	4% (24)	
	*None*		12% (15)	14% (51)	2% (2)	11% (68)	
	*More than 4*		3% (4)	8% (29)	1% (1)	6% (34)	
Daily vegetable portions	*1*	600	56% (67)	29% (106)	41% (49)	37% (222)	p < 0.001
	*2*		28% (33)	15% (55)	37% (44)	22% (132)	
	*3*		2% (3)	4% (13)	18% (22)	6% (38)	
	*4*		2% (2)	2% (7)	2% (3)	2% (12)	
	*None*		12% (15)	47% (168)	1% (1)	31% (184)	
	*More than 4*		0% (0)	3% (11)	1% (1)	2% (12)	
TV viewing (hours/week)		600	12.75/18.00/28.00	7.75/15.00/24.00	9.00/11.00/13.00	9.00/14.00/23.00	p < 0.001
Television set at home (n°)		600	2.00/3.00/3.00	1.00/1.00/2.00	2.00/2.00/2.00	1.00/2.00/2.00	p < 0.001
IBAI score		600	4.00/ 8.00/14.00	7.00/11.00/16.00	8.75/16.50/24.00	7.00/11.00/17.00	p < 0.001

Values for the categorical variables are expressed as a percentage (absolute numbers in parentheses) and those for continuous variables as the median (I quartile / median / III quartile).

### Overall energy intake of children

The median value for energy intake was 234.08 Kcal, roughly corresponding to a median of 2 snacks for each child. The median values for energy intake and glycaemic load are presented in Table 3. The energy intake recorded in each country was not significantly different among the three countries.

**Table 3:** Energy intake and glycaemic load by country

	**Argentina**	**Brazil**	**Mexico**	**Total**
	**(N = 120)**	**(N = 360)**	**(N = 120)**	**(N = 600)**
Energy intake (kcal)	52.73/155.05/233.40	194.70/250.70/373.19	127.39/214.19/328.64	122.49/234.08/349.48
Glycaemic load	2.87/8.45/12.72	10.61/13.66/20.34	6.94/11.67/17.91	6.68/12.76/19.05

Values are I Quartile/Median/III Quartile.

### Effect of toy alone or combined with TV viewing and advertising

Data on the energy intake for the specific study factor subgroups are presented in Table 4. No significant associations between energy intake and the toy being present were found (p = 0.285). No significant associations were found with movie exposure and advertising exposure (p = 0.393).

**Table 4:** Overall energy intake (Kcal) according to the study factors

**Study Factors**	**NoFilmNoSpot**	**FilmNoSpot**	**FilmLowSpot**	**FilmMediumSpot**	**FilmHighSpot**	**Total**
**NoToy**	60	60	60	60	60	300
Median (I-III quartile)	229.45 (11.49-335.31)	239.26 (116.09-348.26)	234.08 (185.16-353.30)	235.71 (128.62-348.80)	235.71 (113.36-348.39)	234.35 (117.99-348.26)
**Toy (N)**	60	60	60	60	60	300
Median (I-III quartile)	297.99 (215.55-388.59)	231.63 (132.16-350.16)	237.35 (170.59-348.12)	220.18 (114.31-349.48)	240.35 (121.94-353.98)	233.53 (133.25-351.66)
**Total (N)**	120	120	120	120	120	600
Median (I-III quartile)	235.99 (128.35-351.53)	232.44 (119.22-348.39)	234.90 (176.17-348.53)	232.44 (116.22-349.48)	237.89 (116.22-350.57)	234.08 (122.49-349.48)

The interaction between the variables Toy and TV was also assessed. The highest energy intake was recorded in the Toy-NoFilmNoSpot group, but the interaction was not significant. The crude effect of the variable Toy can be observed in the NoFilmNoSpot subgroup, with no significant difference between the two groups (Table 4).

After adjustment for all of the potential confounding factors described, no significant association was confirmed in terms of energy intake between the variables Toy or TV viewing or their interaction. All data are presented in Table 5.

**Table 5:** Significance of the factors Toy, TV, advertising and their interactions after adjustment for several potential confounding factors

	**TOY**		**Filmspot**					**TOY: Filmspot**				
	**b**_**g**_	**P-value**	**b**_**TVNs**_	**b**_**TVLs**_	**b**_**TVMs**_	**b**_**TVHs**_	**P-value**	**B**_**TVNs:T**_	**b**_**TVLs:T**_	**b**_**TVMs:T**_	**b**_**TVHs:T**_	**P-value**
**Base**	53.92 (28.93)	0.285	−11.19 (28.93)	18.08 (28.93)	6.46 (28.93)	−0.74 (28.93)	0.665	−38.03 (40.92)	−78.37 (40.92)	−55.22 (40.92)	−28.67 (40.92)	0.393
**Base + M1**	54.30 (28.29)	0.482	−0.73 (28.32)	24.05 (28.79)	10.00 (27.93)	−10.29 (28.24)	0.694	−51.16 (40.29)	−87.80 (40.79)	−73.77 (40.46)	−17..12 (40.09)	0.159
**Base + M1 + M2**	17.56 (39.07)	0.799	−36.47 (37.79)	−20.97 (39.91)	−1.26 (36.52)	−35.87 (38.30)	0.340	−25.68 (53.42)	−41.31 (59.13)	−44.22 (54.65)	42.55 (54.48)	0.486
**Base + M1 + M2 + BA**	17.48 (39.19)	0.497	−37.16 (37.92)	−20.99 (39.97)	−1.45 (36.58)	−36.17 (38.37)	0.342	24.49 (53.65)	−40.91 (59.24)	−43.91 (54.74)	42.43 (54.57)	0.497
**Base + M1 + M2 + BA + M3**	14.99 (38.99)	0.456	−40.26 (37.87)	−16.37 (39.89)	−5.55 (36.63)	−37.20 (38.29)	0.339	−23.34 (53.52)	−48.51 (59.12)	−44.89 (54.57)	44.70 (54.38)	0.428
**Base + M1 + M2 + BA + M3 + M4**	5.51 (39.81)	0.460	−43.57 (39.28)	−14.29 (40.77)	−14.05 (37.46)	−42.52 (40.29)	0.346	−13.68 (54.87)	−39.93 (60.39)	−33.77 (55.68)	47.78 (56.61)	0.932

Models have been selected using the AIC criterion (p-value for staying in the model 0.25). The final model used 7 degrees of freedom for the design variables plus 30 degrees of freedom for the selected additional variables. Cells are p-values related to the variables indicated in the columns and are underlined when significant.

Base: Blocking + TOY + filmspot + TOY: filmspots.

M1: BMI + breast feed + hours/week TV + physical activity (hours/week).

M2: BMI father + BMI mother + number brothers + numbers of sisters.

BA: IBAI - Score.

M3: Number of rooms in the house + number of TV in the house + educational level of the mother.

M4: breakfast in the morning + fruit portions/day + vegetables portions/day.

### High consumer

The top quartile of energy intake corresponded to 349.48 Kcal. This value has been chosen as the cut-off to identity the subgroup of the 150 children with the highest energy intakes during the experimental session. Table 6 shows the main characteristics of these “high consumer” children. At the multivariable analysis level, no significant associations were found for the experimental factor Toy (p-value 0.486), the factor Film Exposure (p-value 0.218) or their interaction (p-value 0.996) with the “high consumer” status. The only variable associated with this status turned out to be the country of the children. Taking Argentina as a reference, the odds ratio (OR) was 6.01 for Brazil (95% C.I. 2.94-12.28) and 2.92 for Mexico (95% C.I. 1.29-6.62).

**Table 6:** Characterization of the high consumers according to the main study variables

	**N**	**Low**	**High**	**Combined**	**p-value**
		**(N = 450)**	**(N = 150)**	**(N = 600)**	
**Country: Argentina**	600	25% (111)	6% (9)	20% (120)	<0.001
**Brazil**		54% (242)	79% (118)	60% (360)	
**Mexico**		22% (97)	15% (23)	20% (120)	
**Toy**	600	48% (218)	55% (82)	50% (300)	0.187
**Gender: Male**	600	49% (219)	54% (81)	50% (300)	0.258
**Age class: Y7-10**	600	46% (207)	62% (93)	50% (300)	<0.001
**FilmSpot: NoFilmNoSpot**	600	20% (89)	21% (31)	20% (120)	0.979
**FilmNoSpot**		20% (92)	19% (28)	20% (120)	
**FilmLowSpot**		20% (91)	19% (29)	20% (120)	
**FilmMediumSpot**		20% (90)	20% (30)	20% (120)	
**FilmHighSpot**		20% (88)	21% (32)	20% (120)	
**Child BMI**	600	14.8800/16.3450/18.4475	15.1200/16.4900/18.0325	14.9125/16.3650/18.3475	0.86
**Breast-feeding**	553	79% (330)	86% (116)	81% (446)	0.189
**Bottle-feeding**		12% (51)	7% (10)	11% (61)	
**Mixed-feeding /breast + bottle)**		9% (37)	7% (9)	8% (46)	
**Number of sisters: 0**	600	51% (230)	49% (73)	50% (303)	0.106
**1**		36% (160)	31% (47)	34% (207)	
**2**		9% (41)	12% (18)	10% (59)	
**3**		3% (13)	4% (6)	3% (19)	
**4**		1% (5)	1% (2)	1% (7)	
**5**		0% (0)	1% (1)	0% (1)	
**6**		0% (1)	2% (3)	1% (4)	
**Number of brothers: 0**	600	48% (216)	44% (66)	47% (282)	0.281
**1**		35% (157)	35% (52)	35% (209)	
**2**		11% (50)	15% (23)	12% (73)	
**3**		5% (22)	4% (6)	5% (28)	
**4**		1% (4)	1% (1)	1% (5)	
**6**		0% (0)	1% (1)	0% (1)	
**8**		0% (1)	0% (0)	0% (1)	
**Weekly TV hours**	600	9.0/14.0/22.0	9.0/13.5/23.0	9.0/14.0/23.0	0.786
**Number of TVs in the house**	600	1/2/2	1/1/2	1/2/2	0.001
**Breakfast before school: 2–3 times per week**	600	5% (22)	5% (8)	5% (30)	0.27
**3–4 times per week**		8% (35)	3% (5)	7% (40)	
**never**		9% (39)	11% (16)	9% (55)	
**every day**		79% (354)	81% (121)	79% (475)	
**Number of fruit portions per day: 1**	600	38% (172)	35% (53)	38% (225)	0.867
**2**		31% (140)	34% (51)	32% (191)	
**3**		9% (41)	11% (17)	10% (58)	
**4**		4% (18)	4% (6)	4% (24)	
**none**		12% (54)	9% (14)	11% (68)	
**more than 4**		6% (25)	6% (9)	6% (34)	
**Number of vegetables portions per day: 1**	600	38% (171)	34% (51)	37% (222)	0.271
**2**		23% (104)	19% (28)	22% (132)	
**3**		5% (24)	9% (14)	6% (38)	
**4**		2% (7)	3% (5)	2% (12)	
**none**		30% (135)	33% (49)	31% (184)	
**more than 4**		2% (9)	2% (3)	2% (12)	
**Mother’s BMI**	485	22.100/24.460/27.425	23.010/24.735/27.480	22.220/24.540/27.480	201
**Father’s BMI**	340	24.3400/25.8000/27.6800	23.2850/25.2150/27.2025	24.2200/25.7450/27.5775	0.05
**IBAI score**	600	7/10/16	8/13/18	7/11/17	0.006

Values for the categorical variables are expressed as a percentage (absolute numbers in parenthesis) and those for the continuous variables as the median (I quartile / median / III quartile).

## Discussion

The first hypothesis that toys might have an effect on increasing energy intake in children was not supported by our results. The median consumption was not significantly different in the two subgroups, Toy and NoToy, and this result was confirmed in all of the countries studied. The second aim of the study was to assess a potential boosting effect of TV viewing on the mere gadget effect in increasing energy intake. The results did not confirm this second hypothesis either. Similar conclusions can be made when considering the influence of advertising, which showed no effect, even at an increasing level of exposure, on energy intake in any of the observed countries. Given the rejection of both hypotheses, the authors of this study performed an in-depth evaluation of the “higher consumer” subgroup to detect any differences in behaviour with regards to the considered objectives of the study. This analysis showed that belonging to a specific country was the only factor influencing an increased energy intake, confirming the conclusions above based on the general sample.

### Interaction with TV and advertising

Previous research on the effects of advertising, including a study by Boyland [26], has shown significant increases in energy intake due to the exposure to specific advertisements, particularly in overweight and obese children, as well as on the effect of TV screening time, acting as a sedentary replacement for physical activity [27]. Based on these results, the authors evaluated the potential effect of these two additional factors on the specified outcome.

The effects of TV viewing and advertising on snack consumption were investigated according to the snack that the children were presented with. The objective was to understand whether the present study was aligned with other studies on the potential role of visual media in increasing total energy intake. Unlike previous studies that reported an association between television viewing and an increase in caloric intake [8,28] and found a positive association between advertising exposure and energy intake [14,29], the children in this particular sample did not increase their intake of snacks while watching TV or when they were subjected to the high-advertisement conditions. Previous studies reported a subtle and potentially far-reaching effect of food advertising on eating behaviours that may occur outside of the participants’ intention or awareness [30]. In our research, the children did not appear to be influenced by the advertisements, even when the interactions were evaluated after adjustment for several potential confounding factors, such as the parents’ BMI, the brand awareness score, the physical activity level or the number of hours of TV viewing. These results, although gathered from an experimental study rather than a real-life scenario, highlighted the fact that, as noted by Goris and colleagues, the contribution of TV advertising of foods and drinks in altering a child’s energy intake differs distinctly by country [31].

The interaction between the presence of the toy and TV and advertisement viewing was also not significant. In the subgroup analysis of those who were not selected for TV viewing (the NoFilmNoSpot group), representing a “TV-free” experimental context, the level of consumption in terms of caloric intake was not significantly related to the presence of the toy.

### The cultural environment

The in-depth analysis performed separately on the three countries under study contributes additional insight beyond the original hypotheses. Although not significantly different, the children’s behaviours were not consistent across the countries. When considering the average energy intake, Argentina presented the lowest levels, with Mexican and Brazilian children eating 1.5 times as much as their Argentinean peers, on average. Country was the only risk factor associated with an augmented intake of snacks; there was no association between the presence of the toy and the increased intake of calories, according to the number of snacks eaten by the child in this *ad libitum* study. Unlike previous studies set in a single country [5,16], children responded with a general lower awareness when questioned on brand, even when considering the highest scores, which were reached in Mexico. Our study, unlike Kopelman’s, [16] included children across a broader age range and focused on 12 specific items related to high energy intake, thus addressing the entire developmental period of children. Differences in the brand awareness of children were also observed within the Latin American setting and were significantly different between Argentina and the other two countries. The median IBAI score observed in Mexican children defined the country as a high brand awareness country, with scores twice as high as those observed in Argentina. Because the present findings challenge previous studies, understanding the influence of culture and setting on different variables and related links to patterns of eating and physical activity, which influence obesity, should be critical in developing public policies and effective clinical interventions to prevent and treat childhood obesity.

### Study Limitations

Although all results were confirmed after adjustment, showing strong consistency, there are several limitations to the study. First, these results refer to an experimental setting and need to be validated in real-life scenarios. However, the choice of an experimental setting allowed us to eliminate a wide variety of biases that could decrease the power of the study. Second, the children could possibly have been aware that the study related to food/body weight because, for logistical reasons, the anthropometric measures were taken before the experimental session. Additionally, the children took part in the study by sitting at a table and being observed by an investigator and interacting with her/him for getting more snacks; all of these factors could potentially have inhibited their behaviour. However, there are no *a priori* reasons to think that this mechanism would have acted selectively on one group more than another. Thus, if bias occurred, most likely it would have been spread out across the groups equally. Third, there was no possibility for the children to choose between different types of snack, given the *a priori* decision to use a single type of snack that was adequate for the research objectives and measurements. The children enrolled in the study might have been limited by the lack of choice in the *ad libitum* snacking setting. Further research involving a broader choice of products will provide a deeper understanding of the children’s behavioural perspectives in afternoon snacking. Finally, the product used in this experimental study is on the border of the definition of gadgets and food because it is a derivation of the traditional “Easter egg”, in which toys and chocolate are present simultaneously (the definition of the product itself).

### Final remarks

Our results showed that the presence of a toy or a toy packaged and sold with a child’s snack did not alter the amount of the item consumed in a Latin American context. In a broader context, our results advocate the need for multi-cultural and cross-cultural research. Indeed, for a full understanding of the association between obesity and its risk factors, in-depth research is necessary in different scenarios to provide the best evidence for decision makers and the basis for prevention in evidence-based strategies rather than focusing on single factors without the recognition of their mutual influence. Moreover, from a public health point of view, effective interventions to improve health and nutritional status need to be grounded on culturally appropriate evidence.

## Competing interests

All authors declare that they have no conflicts of interest.

## Authors’ contributions

DG conceived of the study, participated in the design of the study and performed the statistical analysis; SB participated in drafting the manuscript, CEG helped to draft the manuscript, AC participated in the coordination of the study, GS participated in its design and coordination, RdSA helped in conducting the study, LF participated in its design and coordination and helped to draft the manuscript, IA participated in coordinating the study, NMAdS helped in conducting the study, JD participated in its design and coordination and overviewed manuscript drafting. All authors read and approved the final manuscript.
